# Times

**Published:** 1866-02

**Authors:** 


### Editorial.

### "TIMES."

Not hard times, nor olden times—not even "good old colony times"—nor the "good times coming," but "The Dental Times," for January, which has already come—no, my copy hasn't, (but I hope it is on the way), so I looked at a copy sent to the "Dental Register." And what of the "Dental Times"? Certainly this much, at least:

Being edited by the faculty of the "Pennsylvania College," it has a strong editorial corps, which is slightly numerous, considerably able, and rather experienced. And as they all work, *emeritus* editor as well as the rest, and as the "Times" *are* (?) quarterly, so that they have ample leisure to revise and perfect their work, a good, strong dignified, sober-looking, but racy journal is the result. As a journal, it is thoroughly original, its matter mostly coming from the pens of its editors; yet it finds room for a good article from an outsider, when furnished. Its printers, too have the good taste to make it look a good deal like the "Register;" hence it makes a fine appearance. The readers of the "Register"—and, indeed, anybody else—can obtain a copy at the rate of one dollar, per annum, by addressing Prof. T. L. Buckingham, Philadelphia.

The "G. T. B." editor has made a discovery, and announces his gratification that his "old friend, Dr. George Watt," is again in the editorial chair of the Register; but if he will examine the "chair" closely, he will find that he labored under an optical delusion, induced, perhaps, by a simple accident, the printers, from force of habit, placing a "W." article among the editorial. But I am glad to be able to work for any department of the Register—glad, indeed, that I am alive; and it affords some gratification that I have *friends* who join me in the sentiment. And, so, with my best wishes for the success of the "Times," I am

